# Balancing the books of nature by accounting for ecosystem condition following ecological restoration

**DOI:** 10.1038/s41598-024-62137-5

**Published:** 2024-05-18

**Authors:** Tina Parkhurst, Rachel J. Standish, Suzanne M. Prober, Halina Kobryn, Michael Vardon

**Affiliations:** 1 School of Environmental and Conservation Sciences, Murdoch University, Murdoch, 6150 Australia; 2 CSIRO Environment, Canberra, 2601 Australia; 3 Fenner School of Environment and Society, Australian National University, Canberra, 2601 Australia

**Keywords:** Ecosystem accounting, Ecological condition indicators, Kunming-Montreal Global Biodiversity Framework, Natural capital accounting, Nature Repair Act (2023), Nature Repair Market, System of Environmental-Economic Accounting, Environmental economics, Restoration ecology, Ecology

## Abstract

Demand for ecological restoration of Earth’s degraded ecosystems has increased significantly since the adoption of The Kunming-Montreal Global Biodiversity Framework in December 2022, with target 2 aiming to ensure that at least 30% of degraded ecosystems are under effective restoration by 2030. More recently, in December 2023, the Australian Parliament introduced the Nature Repair Act, which establishes a framework for the world’s first legislated, national, voluntary biodiversity market. How can the effectiveness of these ambitious targets be measured? Natural Capital Accounting (NCA) provides a framework to measure changes in ecosystem condition that is applicable across ecosystems and potentially catalogue effects of restoration interventions to drive investment, improvement to practice, and ultimately, to better protect the Earth's ecosystems. However, the framework has not been tested in this context. In this progressive approach, we populated the leading global NCA framework with ecological data to quantify changes in ecosystem condition after restoration. In principle, NCA is fit for purpose, however, methodological refinements and ecological expertise are needed to unlock its full potential. These tweaks will facilitate adoption and standardisation of reporting as efforts ramp up to meet ambitious global restoration targets.

## Introduction

There is a global biodiversity extinction crisis due to the exploitation and destruction of native ecosystems and the impacts of climate change^1^. The depletion of natural resources in the last 70 years has altered and damaged ecosystems more rapidly and extensively than at any other time in human history^1–3^. Whilst these changes made to ecosystems have led to substantial economic growth and increased people’s well-being in some parts of the world, they have amplified poverty and depleted resources for others^4,5^. Moreover, corporations, investors and governments benefiting greatly from the primary resources that nature provides have not been required to pay for, nor repair, the cumulative damage to natural capital^4^.

Ecological restoration has the potential to halt or reverse damaging effects the exploitation of natural resources has caused^6–8^. Indeed, reforestation, when executed with ecological integrity (sensu^9^, has been highlighted as one of the key climate change and land degradation mitigation actions^10–12^. As such, numerous international agreements (e.g., Global Biodiversity Framework, Bonn Challenge, UN Convention to Combat Desertification, Paris Agreement, International Blue Carbon Initiative, Great Green Wall) have pledged to restore billions of hectares of degraded terrestrial habitat, as well as degraded aquatic and marine habitats, involving significant restoration activities with ambitious targets (e.g., 30% of degraded ecosystems under effective restoration by 2030, CBD^13^. More recently, the Australian Parliament passed the Nature Repair Act^14^ paving the way for a world-first Nature Repair Market. Yet quantitative methods to determine biodiversity and ecosystem recovery following these substantial investments are lacking^15–17^ and are essential if biodiversity markets are to achieve biodiversity conservation^18^. Moreover, whilst ecological restoration has potential to remediate the impacts of climate change and environmental degradation, restoration interventions often fall short of achieving full ecosystem recovery^19,20^. In addition, while some restoration outcomes are immediate, others take time (i.e., 50+ years)^21^. Therefore, we need a framework that can track changes in ecosystem condition through time whilst ensuring that national and international restoration targets are met^22^.

Natural capital accounting is an approach that could potentially synthesize multiple and varied restoration outcomes to assess ambitious restoration targets. In 2021, the United Nations adopted the System of Environmental-Economic Accounting-Ecosystem Accounting (SEEA-EA) as the international standard for natural capital accounting^23^. This framework systematically arranges biophysical and economic measures to account for the extent and condition of stocks and flows (e.g., ecosystem services) within defined environmental units (e.g., ecosystems) comprising nature capital^23,24^. There are natural capital accounts of ecosystem condition, ecosystem services and their monetary evaluation at national and sub-national level^25–30^, which suggest the approach is fit for purpose. Yet, to our knowledge, the approach has not been used to measure outcomes of ecosystem restoration on natural capital (e.g.,^31^.

**Figure 1:**
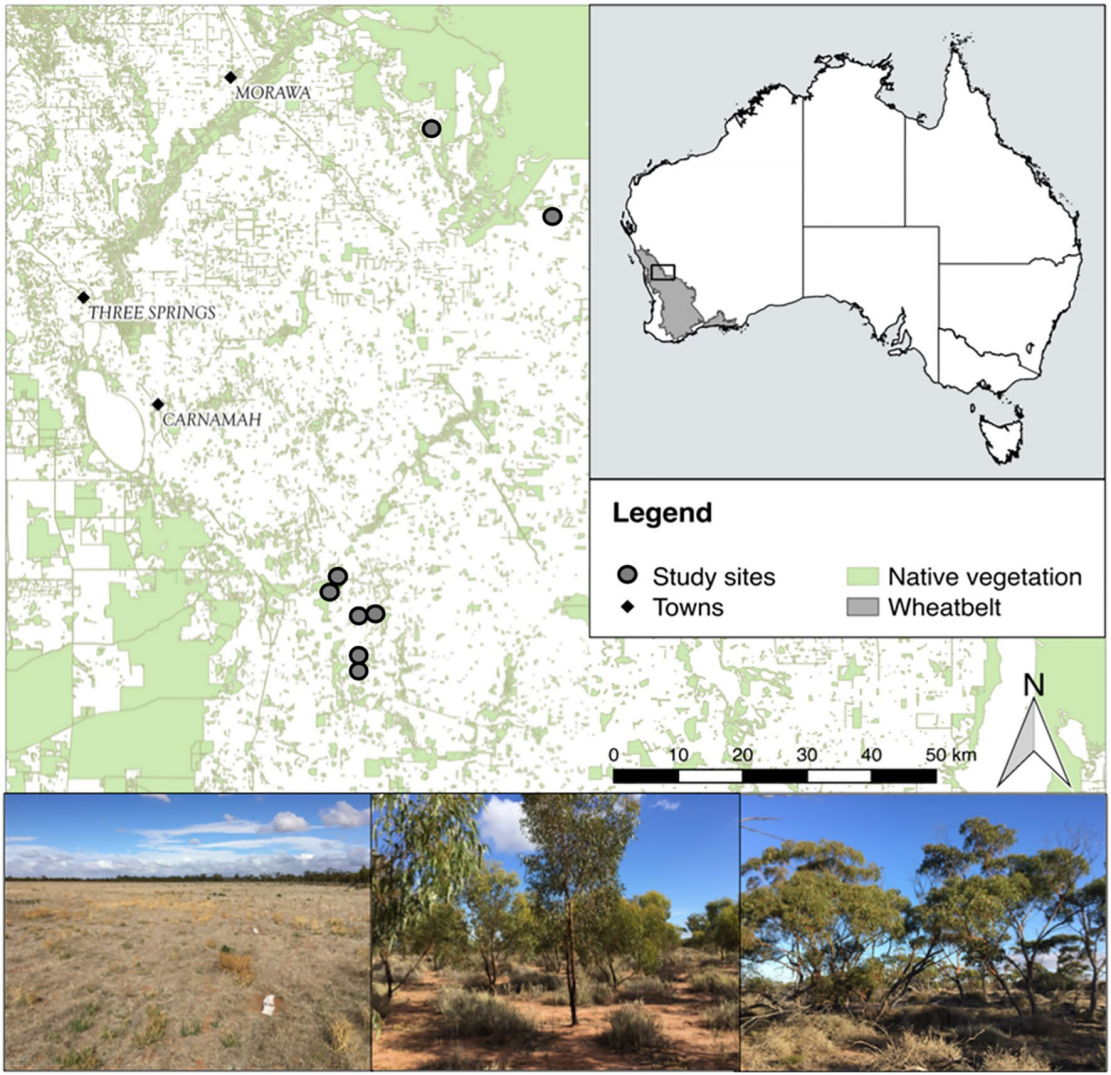
Case study wheatbelt region in western Australia, and images (left to right) showing examples of a fallow cropland/unfavourable reference ecosystem site prior to restoration intervention, the restored site 10 years after planting native trees and shrubs, and the native, intact eucalypt woodland (favourable reference ecosystem). Map created with QGIS version 3.32.1-Lima, qgis.org.

Here we present a case study applying before and after restoration data to the SEEA-EA to test its suitability for recording changes in ecosystem condition resulting from ecological restoration. We use an extensive set of abiotic and biotic data collected for an ecological restoration study of abandoned farmland in Australia (Table S1). Restoration was attempted through native tree and shrub planting, to mitigate climate change^32^, and to provide benefits to biodiversity^33^ and to people^34^. Restoration outcomes at the restored sites were measured 10-years after planting and compared with both the reference ecosystem, a nearby native, intact, eucalypt woodland (hereafter ‘favourable reference ecosystem’) and the starting point for restoration, the fallow cropland (hereafter the ‘unfavourable reference ecosystem’,Fig. 1). Our use of the term ‘reference’ is consistent with SEEA-EA terminology^23^.

## Results

Applying the SEEA-EA framework indicated an ecosystem condition improvement of 50% following planting of native woody shrubs and trees on ex-agricultural land (Figs. 1, 3). The abiotic ecosystem condition improved by 24%, comprising an improvement in soil physical condition of 15% and chemical condition of 9% (Fig. 3). The biotic ecosystem characteristics condition improved by 26%, which included an improvement in the compositional state by 7%, the structural state by 9% and the functional state by 10% (Fig. 3).

**Figure 2:**
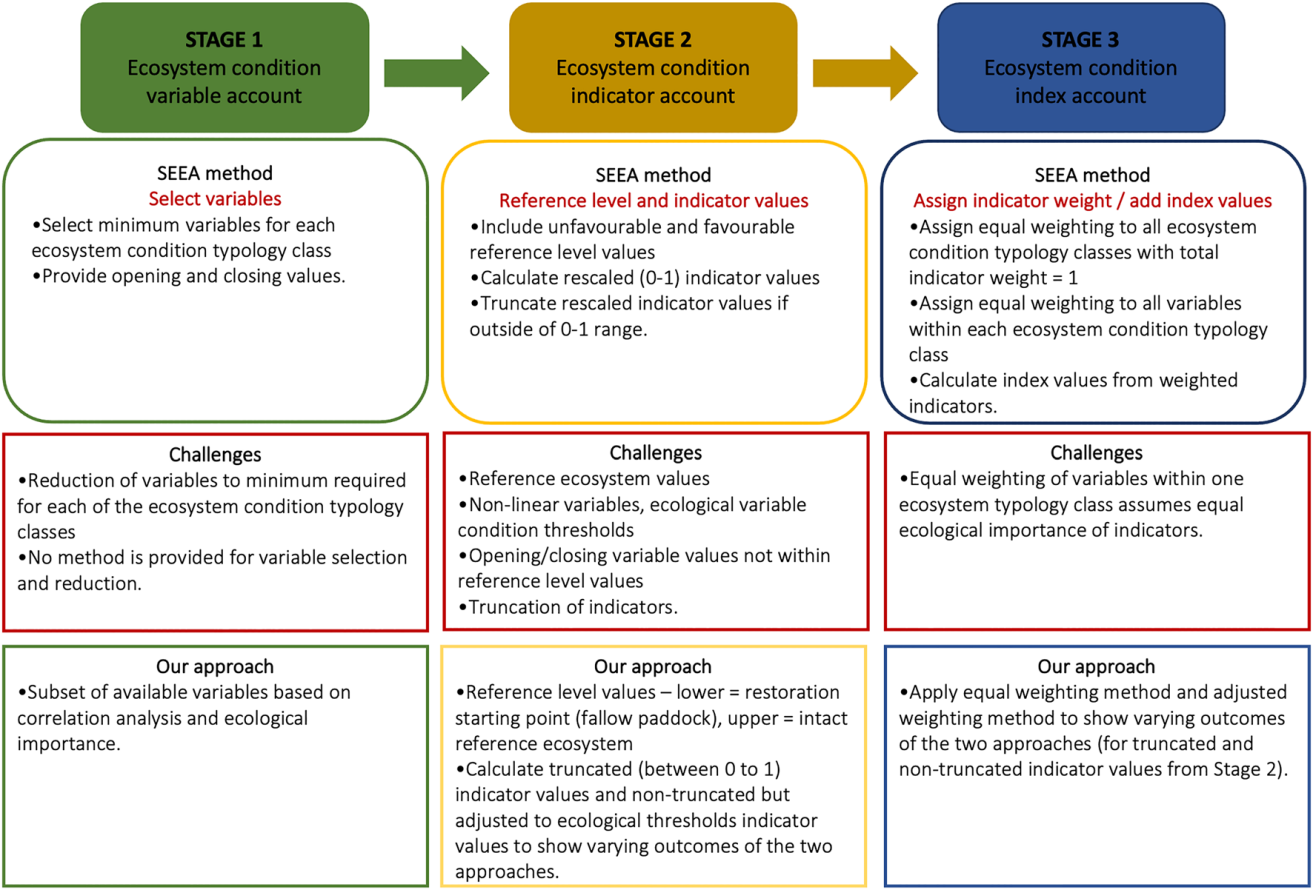
SEEA EA stages 1–3 methods, challenges and approaches applied in this case study.

However, when applying the SEEA-EA to our restoration dataset we found that some of the framework’s methodology was not fit-for-purpose (Fig. 2). Key challenges included truncation of condition values to fit within a condition change of 0–100% only, default equal weighting of condition indicators that may not reflect ecological importance, lack of consideration of ecological thresholds, and the selection of suitable ecosystem reference range values. To overcome these key challenges, we adjusted the methodology proposed by the SEEA-EA and tested three different approaches (Table 1). By omitting the truncation method, the ecosystem condition improved by 46% (Table 1A); equally when indicator weights were adjusted (Table 1B). Differences for the individual condition indicators were minor (e.g., biotic characteristics, Table 1B). Adjusting indicator weights and truncating the condition values to the 0–100% scale, showed an overall ecosystem condition improvement of 49% (Table 1C).

**Table 1:** Summarized condition indices account showing changes in opening and closing condition values following restoration interventions for SEEA-EA approach and the adjusted approaches we tested.

Condition indices account	SEEA-EA approach	Tested approaches in case study		
		A	B	C
	Equal indicator weight, truncation	Equal indicator weight, ecological thresholds, no truncation	Adjusted indicator weight, ecological thresholds, no truncation	Adjusted indicator weight, ecological thresholds, truncation
Opening condition value	0%	0%	0%	0%
Change in abiotic ecosystem characteristics	24%	22%	22%	23%
Physical state	15%	11%	11%	15%
Chemical state	9%	12%	11%	8%
Change in biotic ecosystem characteristics	26%	24%	24%	26%
Compositional state	7%	7%	6%	5%
Structural state	9%	8%	10%	11%
Functional state	10%	8%	8%	10%
Net change in condition	50%	46%	46%	49%
Closing condition value	50%	46%	46%	49%

## Discussion

Overall, this case study demonstrates how the SEEA-EA can be meaningfully applied to quantify changes in ecosystem condition following ecological restoration. Our contrasting approaches did result in minor differences in condition scores; however, other datasets from studies in different ecosystems or tracking different restoration interventions might yield more contrasting results. Here we explore key challenges of the framework around variable selection, reference ecosystem level values, non-linear variables and ecological condition thresholds, and weighting of variables. We discuss our proposed solutions to extend the framework, including an elaboration of the ecosystem condition accounts to capture changes in ecosystem condition following unassisted recovery or restoration interventions (Table 2).

**Table 2:** Proposed ecosystem condition account for inclusion of human-assisted and unassisted ecosystem recovery.

Extended ecosystem condition account
Opening condition value
Human-assisted additions in abiotic and biotic ecosystem characteristics
Physical and chemical state
Compositional, structural and functional state
Unassisted additions in abiotic ecosystem characteristics
Physical and chemical state
Compositional, structural and functional state
Human-assisted reductions in abiotic and biotic ecosystem characteristics
Physical and chemical state
Compositional, structural and functional state
Unassisted reductions in abiotic ecosystem characteristics
Physical and chemical state
Compositional, structural and functional state
Net change in abiotic ecosystem characteristics
Net change in biotic ecosystem characteristics
Net change in condition
Closing condition value

### Selection of variables

The selection of suitable ecological variables or indicators is critical when measuring change in ecosystem condition. For changes in ecosystem condition following restoration, consideration will need to be given to variables that are indicative of overcoming key barriers to ecosystem recovery depending on disturbance type such as abiotic and biotic thresholds to restoration in agricultural landscapes. For example, we included variables for soil nutrient and soil physical properties to indicate changes in these key threshold metrics in old field restoration^35^. In addition, variables tracking change of condition towards the target (usually favourable reference ecosystem) state are essential (e.g., restoration of a woodland should include variables measuring tree species richness and cover)^6,36^. Furthermore, variables that are indicators of the same ecological features and are correlated can be removed (e.g., in our study we removed leaf litter cover as it was highly correlated with tree cover). In the absence of an extensive dataset covering a range of suitable abiotic and biotic indicators as presented in this case study, conceptual models of ecosystems^37^ and their transitioning states^38^ are useful to define appropriate variables to measure change in ecosystem condition following restoration activities. Doing so will provide guidance for data collection prior to creating ecosystem condition accounts and focus on identifying key ecosystem characteristics and functions as well as pathways to recovery.

### Reference ecosystem values and ecological condition thresholds

The SEEA-EA recommends that the range between the unfavourable and favourable reference ecosystem levels for all variables includes an unfavourable (e.g., ecosystem collapse) and favourable (e.g., native, intact ecosystem) condition value. In addition, it states that the unfavourable reference ecosystem levels are often the natural zero value of the variable. However, a zero value for a natural state is not always appropriate for the unfavourable reference ecosystem level because abiotic and biotic features are often present at measurable levels in modified ecosystems (i.e., fallow croplands). We chose the fallow cropland as the unfavourable reference ecosystem level (i.e., ‘collapsed woodland’), to scale the opening value to zero to assess relative change from a common starting point for restoration (i.e., fallow cropland, Table S2). In addition, the SEEA-EA focuses on linear variables (i.e., linear increase equals better condition), and provides limited guidance on non-linear dynamics of variables (i.e., threshold dynamics^39^. For example, both high and low pH can be problematic, and optimal pH more often lies towards the middle of the scale. The truncation of the variables to a dimensionless scale between 0 and 100% is a simple approach to resolving the non-linearity issue. However, this approach implies that exceeding favourable or declining further past unfavourable reference ecosystem levels has no (negative or positive) implications for ecosystem condition. Resolving threshold dynamics is more difficult than for linear dynamics as it requires detailed ecological knowledge for the variable and ecosystem under consideration.

In our case study a negative change in condition scores for individual variables means that the closing value (the restored site average) is not within the reference ecosystem value, and lower than the favourable reference ecosystem value. In ecological terms it can mean that (a) the closing value indicates a lowering of condition (i.e., degradation) and therefore unsuccessful restoration, or (b) the closing value is within an optimum range of the favourable reference ecosystem (e.g., 50–150%^40–42^. Distinguishing between these possibilities is critical because (a) infers restoration failure whereas (b) does not. This means defining meaningful ecological change for each variable in the account. For example, a woodland has an optimum range of 30% to 70% tree canopy cover^43^, any values below or above this threshold signal a worsening in condition. The issue with truncation is that the closing value cannot be outside the favourable and unfavourable reference ecosystem values, hence truncation could conceal lowering of the condition score for some variables. In ecological terms, there are scenarios where the closing balance of a particular variable could be higher than the favourable reference ecosystem condition value, which may indicate that the ecosystem is moving towards a degraded state (e.g., nutrient enrichment) rather than the favourable reference state. In our study the overall non-truncated condition score was only 4% lower than the truncated score, so of no real ecological consequence, however other datasets may yield different, ecologically significant results. Therefore, truncation of the condition scores outside the reference ecosystem levels requires specific ecological consideration and should not be a standard approach. Ecological expertise is required to define appropriate reference ecosystem levels and condition thresholds for meaningful condition accounts.

The assumption of the unfavourable (low) reference ecosystem value to be zero (i.e., lowest value), as suggested in the SEEA-EA framework, does not necessarily suit the assessment of a change in ecosystem condition because the condition of the ecosystem can be low, from an ecological perspective, even though indicators have low or high values as discussed above. The indicators would be ecologically accurate if the unfavourable reference ecosystem value equated to the collapse of the ecosystem—where the ecosystem is transformed unrecognisably due to loss of key biotic and abiotic features^44^. This is referred to as ecosystem conversion in the SEEA EA (para. 4.23^23^. To define the unfavourable state of collapse, the process of defining suitable reference ecosystem levels would necessarily include the definition of collapse of the ecosystem’s key characteristics. A framework that has purposefully defined ecosystem collapse is the IUCN Red List of Ecosystems^44^. This protocol assesses the risk of ecosystem collapse using five quantitative criteria, including two criteria that are relevant to changes in the ecosystems abiotic and biotic features and processes. Ecosystem collapse here is defined as the ecosystem having undergone a transformation of identity, loss of defining abiotic and biotic features as well as characteristic biota and the replacement by a novel ecosystem^44^. Whilst the novel ecosystem may retain some of the features of the original ecosystem, their relative abundances, composition, structure and perhaps functions will be different^44^.

In our case study, a eucalypt woodland ecosystem would be considered ‘collapsed’ if key biotic features such as eucalypt overstory as well as shrubs and herbaceous understory were absent and unlikely to re-colonise (e.g.,^45^. While novel, the collapsed state can however support the growth of other plants (e.g., weeds), and invertebrate populations (e.g., ants, termites), and while classified as an unfavourable, collapsed state, not equate to zero values for all abiotic and biotic ecosystem characteristics. While quantifying the collapsed state may improve capacity to interpret the NCA, it is not straightforward. Indeed, defining ‘zero’ as the starting point for change may be preferrable when it is difficult to ascribe a more ecological meaningful score. Regardless of the approach taken to define unfavourable and favourable reference ecosystem conditions, we recommend careful ecological interpretation to define the range of reference ecosystem states for accountancy purposes. Quantitatively defining the collapsed state of the key abiotic and biotic characteristics of the ecosystem would therefore provide a suitable method to select unfavourable reference ecosystem level indictor values. Whilst the difference in ecosystem condition in this case study is minor between the truncated and non-truncated approach, this might not be the case for other ecosystems. We recommend carefully defining reference ecosystem states using expert ecological knowledge despite the challenge to quantitatively define collapse thresholds for a range of ecosystem indicators^46^.

### Weighting of indicators

Some indicators may influence the condition of the ecosystem more than others because their presence can have significant impacts. For example, in tree dominant ecosystems, old-growth, large trees are a key biotic characteristic of the vegetation structure and provide key structural support and resources and habitat features for other species, below and above ground. Old growth trees are very difficult to replace once lost from an ecosystem and therefore highly valuable^42,47^. Hence under the habitat hectares approach for example, they are given a weight of 10%, which is twice as much as tree canopy cover^42^. Another example are exotic plant species, which influence biomass fuel and litter characteristics, therefore changing ground cover structure, fuel loads, fauna habitat characteristics and recruitment potential of native flora species (e.g.,^48^, and are reflective of novel or collapsed ecosystem states. Similarly, altered soil nutrient concentrations can prevent the establishment and persistence of native flora species and influence soil biological processes^49^. Therefore, exotic plant cover and soil nutrient concentrations may be weighted more highly than other variables.

In our study the difference between weighted and non-weighted (Table 1) ecosystem condition scores was negligible (i.e., compositional state condition degreased by 1%, and structural state condition increased by 2%). However, other studies may yield different results, and warrant higher proportional weighting of key ecosystem condition indicators within the overall condition score^42^. However, more guidance is needed on describing key ecosystem characteristics and the rationale of assigning adjusted weighting according to their importance in assessing ecosystem condition. Further research is therefore needed to outline an approach for indicator weighting that is ecologically meaningful within and among ecosystem types.

### Elaboration of the ecosystem condition account

The ecosystem condition accounts presented in the SEEA-EA do not differentiate changes in condition due to human-assisted ecological restoration from unassisted ecosystem recovery. This is unlike accounts based on ecosystem extent. We propose an elaboration of the ecosystem condition accounts to align the ecosystem extent and condition accounts and make the impact of human intervention clearer to the users of accounts, particularly investors. The alignment of ecosystem extent and condition accounts would assist with distinguishing ecosystem conversion (i.e., from one ecosystem to another), from changes in ecosystems condition. It may also help to incentivise restoration efforts—if these are based on quality (condition) as well as quantity (extent).

Table 2 presents a proposed elaboration of the SEEA-EA “Ecosystem condition account (condition indices) for multiple ecosystem types” (p. 104,^23^. To populate Table 2, the changes in condition need to be attributed to unassisted and human-assisted causes. Methods to do this need development. This could include pairing restored sites with ‘control’ sites in similar opening condition but without restoration activity to distinguish between the two processes to measure restoration impact (e.g.,^50,51^. If the condition accounts are paired with spatially referenced environmental protection and resource management accounts of the SEEA Central Framework^52^, then measures of economic efficiency (i.e., return on investment in restoration) could be derived. Refining the framework, including the elaboration of the ecosystem condition account attributing changes to human intervention will provide a more ecologically sound and integrated approach to calculate ecosystem condition changes following ecological restoration, offering guidance to governments and businesses interested in using SEEA-EA following restoration efforts at local and regional scales. This includes prioritizing efforts towards ecosystems that require intervention for recovery rather than the ecosystems that appear to be recovering, in acceptable timeframes (i.e., by 2050), without this assistance.

## Materials and methods

### Ecosystem

The ecological restoration study was conducted in the Western Australian Wheatbelt (Fig. 1) with the aim to restore York gum woodlands, a sub-vegetation community of the *Eucalyptus* woodlands ecosystem. Intact York gum woodlands are comprised of a highly diverse annual and perennial forb layer, with sparse perennial grasses (e.g., *Austrostipa* spp.) and succulent shrub species (e.g., *Maireana* spp., *Atriplex* spp.) in the vegetation understorey.

York gum woodlands were once expansive and were dominated by *Eucalyptus loxophleba* Benth. and *Acacia acuminata* Benth. Clearing of native vegetation for mixed farming began in the 1900s and continues to date. Approximately 90% of the native vegetation in the landscape has been removed and remaining patches are small and highly fragmented^53,54^. Remnant patches of Western Australian *Eucalyptus* woodlands within the wheatbelt region are now listed as a threatened ecological community and a ‘priority place’ under the Australian Governments Threatened Species Action Plan for 2022–2023^55^. The once wooded landscape is now predominantly comprised of annual crop and grazing lands, classified under the Global Ecosystem Typology as ’Intensive land-use biome- Annual croplands (T7.1), with only small pockets of *Eucalyptus* woodlands (Savannas & Grasslands—Temperate woodlands (T4.4))^56,57^.

### Data

We used abiotic and biotic data collected in a field study in 2017^33,58–60^ assessing restoration outcomes to create the condition accounts. The field study was stratified into three restoration treatments: 1—a fallow cropland, indicative of the restoration starting point and unfavourable reference ecosystem condition state, 2—a previously cropped field, planted with native woody tree and shrub species (*Eucalyptus, Acacia* and *Melaleuca* spp.) approximately 10 years before data collection, and 3—an intact, favourable reference ecosystem site (*Eucalyptus* woodland), indicative of the desired restoration target condition (Fig. 1). Abiotic data comprised physical and chemical soil variables (Table S1). Biotic variables included compositional data for flora and invertebrate fauna, structural vegetation and ground cover information and data describing ecosystem functioning such as soil nutrient availability, decomposition processes and functional groups of invertebrate fauna. Further details on study location, experimental design, data collection methods and results for abiotic and biotic variables are published (and^33,58–60^.

We classified data according to the SEEA-EA Ecosystem Condition Typology (ECT) into groups and classes outlined in Table S1. We did not include landscape level characteristics, because the objective of this case study was to test the suitability of the SEEA-EA to record the restoration outcomes as a ‘proof of concept’, rather than a condition account for a particular spatial unit. Following data classification, we prepared condition accounts, outlined in the three-stage process of the SEEA-EA.

### Ecosystem condition accounts

**Figure 3:**
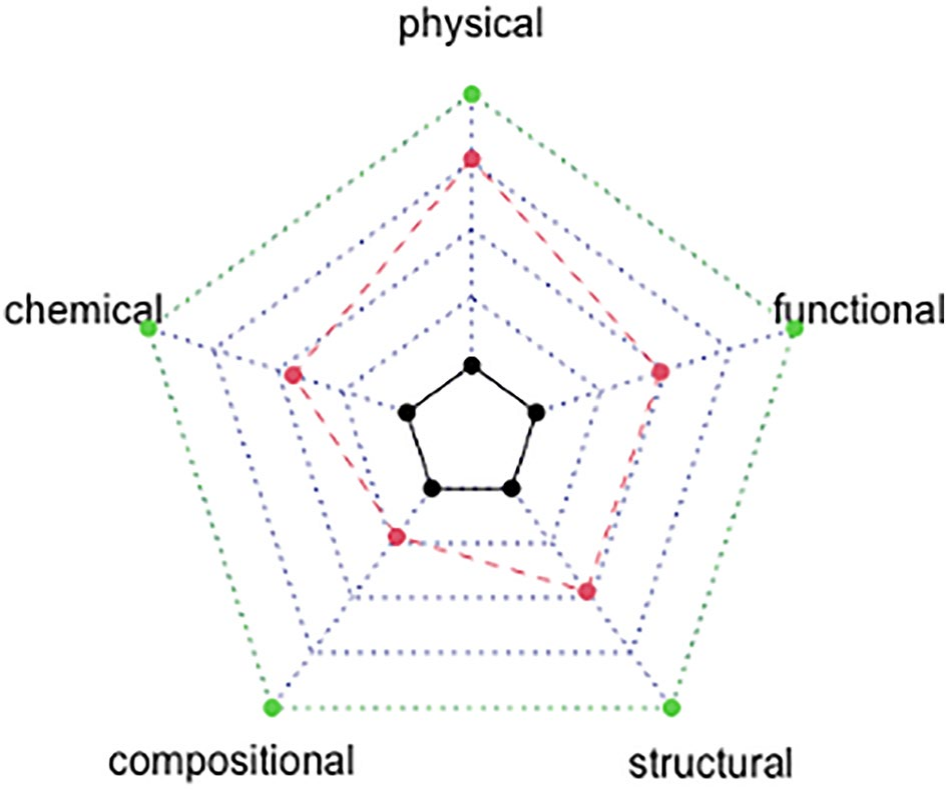
Spider diagram showing average indicator values for each Ecosystem Condition Type class for opening values (black line), closing (restored) values (red line) and intact reference ecosystem values (green line), with truncated and equally weighted indicators (Table 1, SEEA-EA approach).

The SEEA-EA framework uses a three-stage process to calculate ecosystem condition, with each stage producing one set of ecosystem condition accounts. Each stage builds on data and outputs of the previous stage and requires additional information and data to present progressive outputs. Figure 3 below provides an overview of the SEEA-EA method for each of the three stages. When applying the SEEA-EA to our restoration dataset we found that some of the framework’s methodology was challenging to implement. In Fig. 2 we highlight those key challenges as well as our approach to overcome them. The following section provides explicit details on how we applied each condition account stage of the SEEA- EA, the challenges we encountered and our final approach for each stage.

### Stage 1—Ecosystem condition variable account

#### SEEA-EA method

We selected quantitative condition variables to assess ecosystem condition change, these being representative of ecosystem typology classes and determined by data availability. For each of the selected variables, we provided opening and closing values. We used a space-for-time approach^61^ to estimate opening and closing values. The opening value equates to the mean variable measurement value of the fallow paddocks, indicating the restoration starting point (i.e., condition baseline) and the closing value equates to the mean variable measurement values of the 10-year-old planted restoration site (Table S2—Ecosystem condition variable account).

#### Challenges

The SEEA-EA method suggests using as few variables as possible but as many as needed, with a minimum of one variable for each of the ecosystem condition typology classes. Selection criteria for ecosystem characteristics (e.g., key abiotic and biotic characteristics) and their metrics (variables/indicators) are provided and referenced (e.g.,^62^. However, further specific guidance including examples on how to select the most suitable metrics for each of the key ecosystem characteristics relevant to a particular ecosystem would be advantageous.

#### Our approach

To optimise the number of variables, we iteratively reduced the number of available variables from the initial dataset for each of the ecosystem condition typology classes. For each ecosystem typology group (e.g., abiotic or biotic), we created a correlation matrix to identify pairs of variables that were highly correlated (R > 0.7). From these pairs, we retained the most ecologically meaningful variable using our expert knowledge. In addition, we excluded variables that were less ecologically meaningful, for example, we choose to retain only the soil chemistry variables that are directly or indirectly limiting to plant growth or ecosystem processes (i.e., phosphorus, nitrogen, potassium, carbon)^63^.

### Stage 2—Ecosystem condition indicator account

#### SEEA-EA method

This ecosystem condition indicator account compares the change between the opening value and the closing value to a reference ecosystem. The reference ecosystem can be a natural (undisturbed) or anthropogenic system. Reference states of those ecosystems can be selected based on historical, contemporary, least disturbed, or best-attainable condition^64^. In the SEEA-EA, the reference ecosystem levels are to be set to a low (unfavourable) and high (favourable) ecosystem condition value. After selecting the reference ecosystem and unfavourable and favourable values, the resulting indicator values are then rescaled to a uniform dimensionless scale of 0–1 using the following formula:

$${\text{I }} = \, \left( {{\text{V }}{-}{\text{ V}}_{{\text{L}}} } \right)/\left( {{\text{V}}_{{\text{H}}} {-}{\text{ V}}_{{\text{L}}} } \right)$$

where I is the value of the indicator, V is the value of the variable, V_H_ is the high condition score and V_L_ is the low condition score. If the opening and closing values lie outside the reference level values, the resulting indicator values are truncated to fit the required 0 -1 indicator scale.

#### Challenges

##### Selection of reference ecosystem value range

The SEEA-EA method suggests that the reference system value range should be based on unfavourable (e.g., ecosystem collapse) and favourable (e.g., native, intact ecosystem) condition indicators. It is recommended that one of the reference levels can often be set to zero, however this can provide challenges when operationalising this approach. In our case study, the modified ecosystem (i.e., croplands) support a range of abiotic and biotic ecosystem characteristics (e.g., invertebrates and soil nutrients) and cannot be assumed to be of zero value. A definition or assessment process of ecosystem collapse of the system that is to be restored (i.e., eucalypt woodland) would help to define appropriate ecosystem reference values for the unfavourable and favourable condition thresholds.

##### Truncation of opening/closing values outside reference ecosystem value range

The SEEA-EA method assumes that the opening and closing variable values of the ecosystem condition account are within the unfavourable and favourable reference system value range and that it is unlikely that they will be outside that range. In our dataset, 40% of the opening and closing values did not fit within the reference ecosystem values. The SEEA-EA method suggests that in the unlikely event of values being outside the reference level range they need to be truncated to 0 or 1, assuming that the closing value of the ecosystem condition variable account cannot be lower than the unfavourable condition reference value, or higher than the favourable condition reference value, therefore a condition cannot worsen compared to the opening value or increase beyond the favourable condition reference value. These scenarios do not describe the bounds of ecological possibility and potential condition change following restoration.

##### Non-linear variable range of reference ecosystem values

The SEEA-EA recognises that the variables used to measure ecological condition may have non-linear responses to human interventions or natural changes and that different thresholds may apply^20^, paras 5.43 and 5.93). However, the ecosystem condition indicator account methodology focuses predominantly on linear variables.

#### Our approach

##### Selection of reference ecosystem value range

For reference ecosystem values, we include unfavourable (lower) and favourable (high) reference values for all variables from Stage 1. We selected the mean values of the variables measured at the restoration starting point (fallow paddock), as the low (unfavourable) condition score in the ecosystem condition indicator account (Table S3), because the fallow paddock represents the collapsed woodland ecosystem. Similarly, we chose the mean values of the variables measured in the intact woodlands as the high (favourable) condition reference data point in the ecosystem condition indicator account (Table S3). The intact woodlands in our study region are the most ecologically appropriate ecosystem to derive representative ecological condition data and therefore qualify as an appropriate reference system and desired condition indicator^65^. In some cases, the unfavourable value was the high value, and the favourable value was the lower value (e.g., when low bulk density or non-native plant cover values indicate better ecosystem condition than high values).

##### Truncation of opening/closing values outside reference ecosystem value range and non-linear variables

Using the low and high reference level values, we rescaled the ecosystem condition variable values to indicators on a uniform dimensionless scale between 0 and 1, as per SEEA-EA (Table S3). If the initial opening and/or closing values of the ecosystem condition variable account lie outside the low and high reference level values, indicator values are calculated to be higher or lower than 0–1. Therefore, they need to be truncated to fit the rescaled range of 0–1. By doing so, indicator values are re-aligned with the low and high reference level values, and this assumes that the closing value of the ecosystem condition variable account cannot be lower than the unfavourable condition reference value, or higher than the favourable condition reference value (SEEA-EA Sect. 5.3^66^.

As this assumption may not always hold true, we have calculated a separate ecosystem condition indicator account with non-truncated indicator values, but considering ecological meaningful thresholds following existing methodologies ((e.g.,^41^, rather than assuming linearity of variables. Two ecosystem condition indicator accounts are presented to highlight the varying outcomes of the two approaches (Table S3).

### Stage 3—Ecosystem condition index account

#### SEEA-EA method

In the ecosystem condition index account, indicator values of variables from Stage 2 are re-scaled according to an equal weighting of each of the ecosystem condition typology classes (physical, chemical, compositional, structural and functional) and the total indicator weighting for all variables amounts to 1 (100%), (Table S4).

#### Challenges

Equal weighting of indicators may in some instances not be the most ecological meaningful approach, as some indicator might be more important to assessing condition score than others (e.g., cover of exotic plant species). These should be given a higher weighting from an ecological perspective. The SEEA-EA recognises that non-equal weighting may be appropriate, but specific methods for how to apply different weighting systems for indicators of unequal ecological importance is not provided.

#### Our approach

We approached Stage 3 with two methods: (a) we assigned all indicator variables within one ecosystem condition typology class with the same weighting, and (b) we weighted variables within one ecosystem condition typology class according to expert-assessed ecological importance. For abiotic variables, we adjusted weightings to reflect relevance of variables that would impede recovery (e.g., soil phosphorus). For vegetation compositional and structural variables, we assigned weightings in alignment with the ‘Habitat Hectares’ method, an established Australian vegetation assessment framework (Parkes, Newell and Cheal^42^, Table S4). We compare findings using both methods.

### Supplementary Information

Supplementary Information.

## Data Availability

All data are available in the main text or the supplementary materials.
