# Supplementary material for: Balancing the books of nature by accounting for ecosystem condition following ecological restoration
Source: Sci Rep. 2024 May 18;14:11369. doi: 10.1038/s41598-024-62137-5 (PMC11102552; doi:10.1038/s41598-024-62137-5)
Supplement: Supplementary file 1 — Supplementary Information. [file 41598_2024_62137_MOESM1_ESM.pdf]

Supplementary Materials

Balancing the books of nature by accounting for ecosystem condition following ecological restoration

Tina Parkhurst[1]*, Rachel J. Standish[1], Suzanne M Prober[2], Halina Kobryn[1], Michael Vardon[3],

[1]School of Environmental and Conservation Sciences, Murdoch University; Murdoch, 6150, Australia.

[2]CSIRO Environment; Canberra, 2601, Australia.

[3]Fenner School of Environment and Society, Australian National University; Canberra, 2601, Australia

*Corresponding author email: Tina.Parkhurst@murdoch.edu.au,

Background information

The data presented in tables S1-S4 were collected as part of a restoration study (2016-2017) across *Eucalyptus* woodlands in Western Australia. These woodlands are classified as Temperate woodlands in the Global Ecosystem Typology and characterised by a relatively simple structure of tree canopies with a ground layer of grasses and sparce shrubs (*1*). Temperate woodlands are globally distributed across temperate climates, with low to moderate rainfall and warm-season droughts. Across the Western Australian wheatbelt, many *Eucalyptus* woodlands were cleared for farming, mostly cropping and grazing, resulting in a highly fragmented landscape and widespread degradation and loss of integrity of the woodlands. Consequently, *Eucalyptus* woodlands are now listed as a threatened ecosystem under Australian environmental legislation (*2*). Planting native trees and shrubs on degraded and unproductive farmland is a common ecological restoration action in the Western Australian wheatbelt region. For the restoration study, data were collected from fallow croplands, indicating the unfavourable reference ecosystem and the starting point of restoration, the 10-year-old planted sites and intact eucalyptus woodland sites (the favourable reference ecosystem). Detailed descriptions of study location, methods and variables used to characterise soil properties (*3, 4*), vegetation structure and composition (*5*), and ant communities (*6*) are available from other publications.

The System of Environmental Economic Accounting – Ecosystem Accounting (SEEA – EA, (*7*)) consists of a system of integrated ecosystem accounts describing physical and monetary ecosystem extent, condition, services and assets. For this study we focused on changes in ecosystem condition following ecological restoration actions, and populated ecosystem condition accounts, following the SEEA-EA three stage approach. The ecosystem condition accounts organise biophysical information on the condition of ecosystem types. Each stage of the condition accounts produces stand-alone accounts. Moving from one to the next stage progressively increases data requirements and outputs. The ecosystem condition variable account (Stage 1) records opening and closing entries for selected variables for an ecosystem type, grouped by ecosystem condition typology. The ecosystem condition indicator account (Stage 2) builds on the ecosystem condition variable account by rescaling the ecosystem condition variables, which are set against reference ecosystem levels, and then converted to ecosystem indicators. The ecosystem condition index account (Stage 3) provides an aggregated ecosystem condition indicator account to generate summarised information.

Table S1. Data classification into Ecosystem Typology Classes (ETC) as per SEEA-EA following exclusion of correlated variables

Ecosystem Typology Group	Ecosystem Typology Class	Variables
**Group A: Abiotic ecosystem characteristics**	**Class A1. Physical state characteristics**	Soil bulk density
		Soil volumetric water content
		Soil surface hardness
		Soil penetration depth
		Bare ground
	**Class A2. Chemical state characteristics**	Soil organic carbon
		Soil phosphorus
		Soil ammonium
		Soil nitrate
		Soil potassium
		Soil pH ($CaCl_2$)
		Soil EC
**Group B: Biotic ecosystem characteristics**	**Class B1. Compositional state characteristics *(vegetation and invertebrate fauna)***	Total non-native (annual herbaceous) species richness
		Native Tree species richness
		Native Shrub species richness
		Native Grasses species richness
		Native Perennial forbs species richness
		Native Annual forbs species richness
		Shannon-Wiener native flora species diversity index
		Ant (Formicidae spp.) total abundance
		Ant (Formicidae spp.) species richness
	**Class B2. Structural state characteristics (*vegetation)***	Native Tree cover
		Native Shrub cover
		Native Grasses cover
		Native Perennial forbs cover
		Native Annual forbs cover
		Non-native (annual herbaceous) species cover
		Leaf litter cover
		Woody debris (< 10 cm diameter) cover
		Logs (> 10 cm diameter) cover
		Logs (> 10 cm diameter) volume
	**Class B3. Functional state characteristics (*soil functions and ant functional groupings as per Andersen (8)*)**	Soil phosphorus availability
		Soil ammonium availability
		Soil nitrate availability
		Soil potassium availability
		Decomposition (leaves)
		Decomposition (wood)
		Dominant Dolichoderinae - abundance
		Dominant Dolichoderinae - richness
		Generalized Myrmicinae - abundance
		Generalized Myrmicinae - richness
		Opportunists - abundance
		Opportunists - richness
		Subordinate Camponotini - abundance
		Subordinate Camponotini - richness
		Hot-Climate Specialists - abundance

.

Table S2. Ecosystem condition variable account – Stage 1

Table S2 shows the ecosystem condition variable account produced in 'Stage 1' of the condition accounts, and is comprised of the abiotic and biotic characteristics (1) of the Ecosystem Condition Typology Classes, the physical, chemical, compositional, structural and functional sub-states within those classes (2), the corresponding variables (3) and their measurement units (4), as well as opening (5), closing (6) values and the difference between opening and closing values (7). The opening value equates to the mean variable measurement value of the fallow paddocks, indicating the restoration starting point (i.e., condition baseline) and the closing value equates to the mean variable measurement values of the 10-year-old planted restoration site.

Ecosystem Condition Variable Account

IUCN Ecosystem classification
REALM: Terrestrial
BIOME: T4 Savannas & grasslands
Global Ecosystem Typology: T4.4 - Temperate woodlands

SEEA Ecosystem Condition Typology Class		Variable descriptor	Measurement unit	Variable values (observed)		
				Opening	Closing	Change
(1)	(2)	(3)	(4)	(5)	(6)	(7)
**Abiotic characteristics**	Physical state	Soil Bulk Density	cm3	1.60	1.52	-0.08
		Soil Volumetric Water Content	%	8.54	6.91	-1.63
		Soil surface hardness (penetration resistance)	MPa	1.37	1.32	-0.05
		Soil penetration depth	cm	3.26	6.31	3.05
		Bare ground	%	26.67	44.00	17.33
	Chemical state	Soil organic carbon	t C/ha	13.92	13.53	-0.39
		Soil phosphorus	kg/ha	38.40	25.84	-12.56
		Soil ammonium	kg/ha	10.56	5.47	-5.09
		Soil nitrate	kg/ha	34.56	11.55	-23.01
		Soil total nitrogen	t C/ha	1.23	1.12	-0.11
		Soil potassium	kg/ha	460.80	337.97	-122.83
		Soil sulphur	kg/ha	25.58	18.65	-6.93
		Soil pH (CaCl2)	pH level	5.42	5.31	-0.11
		Soil EC	dS/m	0.09	0.17	0.08
**Biotic characteristics**	Compositional state	*Vegetation*				
		Total non-native (annual herbaceous) species richness	number	8.66	9.33	0.67
		Native Tree species richness	number	0.00	1.66	1.66
		Native Shrub species richness	number	1.77	6.77	5.00
		Native Grasses species richness	number	1.88	1.88	0.00
		Native Perennial forbs species richness	number	0.89	1.67	0.78
		Native Annual forbs species richness	number	3.44	3.22	-0.22
		Shannon-wiener native flora diversity	index	1.46	1.81	0.35
		Invertebrates (ants)				
		Total abundance ants	number	189.50	261.13	71.63
		Species richness ants	number	12.63	14.82	2.19
	Structural state	*Vegetation*				
		Native Tree cover	%	0.00	28.67	28.67
		Native Shrub cover	%	2.89	17.50	14.61
		Native Grasses cover	%	3.11	5.83	2.72
		Native Perennial forbs cover	%	0.39	1.11	0.72
		Native Annual forbs cover	%	12.33	3.72	-8.61
		Non-native (annual herbaceous) species cover	%	69.90	23.40	-46.50
		Leaf litter cover	%	0.00	33.11	33.11
		Woody debris (< 10 cm diameter) cover	%	0.11	6.00	5.89
		Logs (> 10 cm diameter) cover	%	0.00	0.22	0.22
		Logs (> 10 cm diameter) volume	m3	0.000	0.003	0.003
	Functional state	*Soil*				
		Soil phosphorus availability	µg/10cm2/3 months	7.00	7.66	0.66
		Soil ammonium availability (NH4)	µg/10cm2/3 months	17.50	7.90	-9.60
		Soil nitrate availability (NO3)	µg/10cm2/3 months	223.90	81.95	-141.95
		Soil potassium availability	µg/10cm2/3 months	359.00	271.65	-87.35
		Decomposition (green tea)	% weightloss	51.66	50.06	-1.60
		Decomposition (roiboos tea)	% weightloss	18.51	17.73	-0.78
		Decomposition (wood)	% weightloss	5.84	6.98	1.14
		Invertebrates (ants)				
		Dominant Dolichoderinae - abundance	number	115.13	201.19	86.06
		Dominant Dolichoderinae - richness	number	2.50	2.82	0.32
		Generalized Myrmicinae - abundance	number	32.63	19.69	-12.94
		Generalized Myrmicinae - richness	number	3.13	2.69	-0.44
		Opportunists - abundance	number	8.63	12.63	4.00
		Opportunists - richness	number	2.00	2.13	0.13
		Subordinate Camponotini - abundance	number	0.25	2.01	1.76
		Subordinate Camponotini - richness	number	0.25	1.76	1.51
		Hot-Climate Specialists - abundance	number	32.88	25.19	-7.69

Table S3. Ecosystem condition indicator account

Table S3 shows the ecosystem condition indicator account produced in ‘Stage 2’ of the condition accounts and builds on the components (1)-(6) of the ecosystem condition variable account, by adding unfavourable/lower (7) and favourable/upper (8) reference ecosystem levels as well as untruncated (9) – (11) and truncated (12) – (13) rescaled indicator values. The unfavourable (lower) reference ecosystem levels correspond to the fallow cropland data (i.e., collapsed ecosystem state) and the favourable (upper) reference ecosystem levels correspond to the intact reference ecosystem data (*Eucalyptus* woodland).

Ecosystem Condition Indicator Account

IUCN Ecosystem classification
REALM: Terrestrial
BIOME: T4 Savannas & grasslands
Global Ecosystem Typology: T4.4 - Temperate woodlands

SEEA Ecosystem Condition Typology Class		Variable descriptor	Measurement unit	Variable values		Reference level values		Indicator values (rescaled) NOT TRUNCATED			Indicator values (rescaled) TRUNCATED TO 0 - 1		
				Opening	Closing	Lower level	Upper level	Opening	Closing	Change	Opening	Closing	Change
(1)	(2)	(3)	(4)	(5)	(6)	(7)	(8)	(9)	(10)	(11)	(12)	(13)	(14)
**Abiotic characteristics**	Physical state	Soil Bulk Density	cm3	1.6	1.52	1.6	1.46	0.00	0.57	0.57	0.00	0.57	0.57
		* Soil Volumetric Water Content	%	8.54	6.91	8.54	7.93	0.00	0.87	0.87	0.00	1.00	1.00
		Soil surface hardness (penetration resistance)	MPa	1.37	1.32	1.37	1.32	0.00	0.91	0.91	0.00	0.91	0.91
		Soil penetration depth	cm	3.26	6.31	3.26	12.58	0.00	0.33	0.33	0.00	0.33	0.33
		* Bare ground	%	26.67	44	26.67	32.44	0.00	0.00	0.00	0.00	1.00	1.00
	Chemical state	* Soil organic carbon	t C/ha	13.92	13.53	13.92	17.37	0.00	0.78	0.78	0.00	0.00	0.00
		Soil phosphorus	kg/ha	38.4	25.84	38.4	8.47	0.00	0.42	0.42	0.00	0.42	0.42
		Soil ammonium	kg/ha	10.56	5.472	10.56	4.89	0.00	0.90	0.90	0.00	0.90	0.90
		* Soil nitrate	kg/ha	34.56	11.55	34.56	22.05	0.00	0.52	0.52	0.00	1.00	1.00
		Soil potassium	kg/ha	460.8	337.97	460.8	302.80	0.00	0.78	0.78	0.00	0.78	0.78
		* Soil pH (CaCl2)	pH level	5.42	5.31	5.42	5.64	0.00	0.00	0.00	0.00	0.00	0.00
		* Soil EC	dS/m	0.09	0.17	0.09	0.09	0.00	0.68	0.68	0.00	0.00	0.00
**Biotic characteristics**	Compositional state	*Vegetation*											
		* Total non-native (annual herbaceous) species richness	number	8.66	9.33	8.66	8	0.00	0.00	0.00	0.00	0.00	0.00
		* Native Tree species richness	number	0	1.66	0	1.44	0.00	1.00	1.00	0.00	1.00	1.00
		Native Shrub species richness	number	1.77	6.77	1.77	10.22	0.00	0.59	0.59	0.00	0.59	0.59
		Native Grasses species richness	number	1.88	1.88	1.88	3.33	0.00	0.00	0.00	0.00	0.00	0.00
		Native Perennial forbs species richness	number	0.89	1.67	0.89	4.33	0.00	0.23	0.23	0.00	0.23	0.23
		* Native Annual forbs species richness	number	3.44	3.22	3.44	7.78	0.00	0.39	0.39	0.00	0.00	0.00
		Shannon-wiener native flora diversity	index	1.46	1.81	1.46	2.49	0.00	0.34	0.34	0.00	0.34	0.34
		Invertebrates (ants)											
		* Total abundance ants	number	189.5	261.13	189.5	187.38	0.00	0.00	0.00	0.00	0.00	0.00
		Species richness ants	number	12.63	14.82	12.63	15.44	0.00	0.78	0.78	0.00	0.78	0.78
	Structural state	*Vegetation*											
		Native Tree cover	%	0	28.67	0	45.56	0.00	0.63	0.63	0.00	0.63	0.63
		Native Shrub cover	%	2.89	17.5	2.89	29.78	0.00	0.54	0.54	0.00	0.54	0.54
		Native Grasses cover	%	3.11	5.83	3.11	7.44	0.00	0.63	0.63	0.00	0.63	0.63
		Native Perennial forbs cover	%	0.39	1.11	0.39	5.88	0.00	0.13	0.13	0.00	0.13	0.13
		Native Annual forbs cover	%	12.33	3.72	12.33	7.28	0.00	0.41	0.41	0.00	1.00	1.00
		Non-native (annual herbaceous) species cover	%	69.9	23.4	69.9	3.055	0.00	0.70	0.70	0.00	0.70	0.70
		Leaf litter cover	%	0	33.11	0	46.44	0.00	0.71	0.71	0.00	0.71	0.71
		Woody debris (< 10 cm diameter) cover	%	0.11	6	0.11	16.67	0.00	0.36	0.36	0.00	0.36	0.36
		Logs (> 10 cm diameter) cover	%	0	0.22	0	8	0.00	0.03	0.03	0.00	0.03	0.03
		Logs (> 10 cm diameter) volume	m3	0	0.003	0	0.18	0.00	0.02	0.02	0.00	0.02	0.02
	Functional state	*Soil*											
		* Soil phosphorus availability	µg/10cm2/3mt	7	7.66	7	1.25	0.00	0.00	0.00	0.00	0.00	0.00
		Soil ammonium availability (NH4)	µg/10cm2/3mt	17.5	7.9	17.5	4.70	0.00	0.75	0.75	0.00	0.75	0.75
		* Soil nitrate availability (NO3)	µg/10cm2/3mt	223.9	81.95	223.9	153.30	0.00	0.53	0.53	0.00	1.00	1.00
		Soil potassium availability	µg/10cm2/3mt	359	271.7	359	166.75	0.00	0.45	0.45	0.00	0.45	0.45
		Decomposition (green tea)	% weightloss	51.66	50.06	51.66	49.83	0.00	0.87	0.87	0.00	0.87	0.87
		Decomposition (roiboos tea)	% weightloss	18.51	17.73	18.51	16.10	0.00	0.32	0.32	0.00	0.32	0.32
		Decomposition (wood)	% weightloss	5.84	6.98	5.84	11.29	0.00	0.21	0.21	0.00	0.21	0.21
		Invertebrates (ants)											
		* Dominant Dolichoderinae - abundance	number	115.13	201.2	115.13	123.94	0.00	0.00	0.00	0.00	1.00	1.00
		Dominant Dolichoderinae - richness	number	2.5	2.815	2.5	2.88	0.00	0.83	0.83	0.00	0.83	0.83
		* Generalized Myrmicinae - abundance	number	32.63	19.69	32.63	36.76	0.00	0.54	0.54	0.00	0.00	0.00
		* Generalized Myrmicinae - richness	number	3.13	2.69	3.13	3.07	0.00	0.88	0.88	0.00	1.00	1.00
		* Opportunists - abundance	number	8.63	12.63	8.63	6.32	0.00	0.00	0.00	0.00	0.00	0.00
		* Opportunists - richness	number	2	2.13	2	1.88	0.00	0.00	0.00	0.00	0.00	0.00
		Subordinate Camponotini - abundance	number	0.25	2.005	0.25	6.19	0.00	0.30	0.30	0.00	0.30	0.30
		Subordinate Camponotini - richness	number	0.25	1.76	0.25	2.94	0.00	0.56	0.56	0.00	0.56	0.56
		Hot-Climate Specialists - abundance	number	32.88	25.19	32.88	13.57	0.00	0.40	0.40	0.00	0.40	0.40

Table S4. Ecosystem condition index account *(Part 1 of 2)*

Table S4 shows the ecosystem condition index account produced in 'Stage 3' of the condition accounts and builds on the components (9)-(14) of the ecosystem condition indicator account, by adding equal indicator weightings (4.1) for each ecosystem condition type class and calculated index values for untruncated and truncated indicator values (7.1-9.1), (7.2-9.2), as well as adjusted indicator weightings (4.2) for untruncated and truncated indicator values (7.3-9.3), (7.4-9.4). The table has been split into two parts for better readability. Please note that due to rounding total sums may slightly vary compared to the sums of the individual characteristics.

Ecosystem condition index accounts														
IUCN Ecosystem classification														
REALM: Terrestrial														
BIOME: T4 Savannas & grasslands			EQUAL INDICATOR WEIGHT											
Global Ecosystem Typology: T4.4 - Temperate woodlands			NOT TRUNCATED						TRUNCATED TO REFERENCE SYSTEM					
SEEA Ecosystem Condition Typology Class		Variable descriptor	Indicator weight	Indicator values (0 - 1)		Index values			Indicator values (0 - 1)		Index values			
				Opening	Closing	Opening	Closing	Change*	Opening	Closing	Opening	Closing	Change*
(1)	(2)	(3)	(4.1)	(5.1)	(6.1)	(7.1)	(8.1)	(9.1)	(5.2)	(6.2)	(7.2)	(8.2)	(9.2)
Abiotic characteristics	Physical state	Soil Bulk Density	0.04	0.00	0.57	0.00	0.02	0.02	0.00	0.57	0.00	0.02	0.02
		Soil Volumetric Water Content	0.04	0.00	0.87	0.00	0.03	0.03	0.00	1.00	0.00	0.04	0.04
		Soil surface hardness (penetration resistance)	0.04	0.00	0.91	0.00	0.04	0.04	0.00	0.91	0.00	0.04	0.04
		Soil penetration depth	0.04	0.00	0.33	0.00	0.01	0.01	0.00	0.33	0.00	0.01	0.01
		Bare ground	0.04	0.00	0.00	0.00	0.00	0.00	0.00	1.00	0.00	0.04	0.04
	Chemical state	Soil organic carbon	0.03	0.00	0.78	0.00	0.02	0.02	0.00	0.00	0.00	0.00	0.00
		Soil phosphorus	0.03	0.00	0.42	0.00	0.01	0.01	0.00	0.42	0.00	0.01	0.01
		Soil ammonium	0.03	0.00	0.90	0.00	0.03	0.03	0.00	0.90	0.00	0.03	0.03
		Soil nitrate	0.03	0.00	0.52	0.00	0.01	0.01	0.00	1.00	0.00	0.03	0.03
		Soil potassium	0.03	0.00	0.78	0.00	0.02	0.02	0.00	0.78	0.00	0.02	0.02
		Soil pH (CaCl2)	0.03	0.00	0.00	0.00	0.00	0.00	0.00	0.00	0.00	0.00	0.00
		Soil EC	0.03	0.00	0.68	0.00	0.02	0.02	0.00	0.00	0.00	0.00	0.00
	Total abiotic					0.00	0.22	0.22			0.00	0.24	0.24
Biotic characteristics	Compositional state	*Vegetation*											
		Total non-native (annual herbaceous) species richnes	0.02	0.00	0.00	0.00	0.00	0.00	0.00	0.00	0.00	0.00	0.00
		Native Tree species richness	0.02	0.00	1.00	0.00	0.02	0.02	0.00	1.00	0.00	0.02	0.02
		Native Shrub species richness	0.02	0.00	0.59	0.00	0.01	0.01	0.00	0.59	0.00	0.01	0.01
		Native Grasses species richness	0.02	0.00	0.00	0.00	0.00	0.00	0.00	0.00	0.00	0.00	0.00
		Native Perennial forbs species richness	0.02	0.00	0.23	0.00	0.01	0.01	0.00	0.23	0.00	0.01	0.01
		Native Annual forbs species richness	0.02	0.00	0.39	0.00	0.01	0.01	0.00	0.00	0.00	0.00	0.00
		Shannon-wiener native flora diversity	0.02	0.00	0.34	0.00	0.01	0.01	0.00	0.34	0.00	0.01	0.01
		Invertebrates (ants)											
		Total abundance ants	0.02	0.00	0.00	0.00	0.00	0.00	0.00	0.00	0.00	0.00	0.00
		Species richness ants	0.02	0.00	0.78	0.00	0.02	0.02	0.00	0.78	0.00	0.02	0.02
	Structural state	*Vegetation*											
		Native Tree cover	0.02	0.00	0.63	0.00	0.01	0.01	0.00	0.63	0.00	0.01	0.01
		Native Shrub cover	0.02	0.00	0.54	0.00	0.01	0.01	0.00	0.54	0.00	0.01	0.01
		Native Grasses cover	0.02	0.00	0.63	0.00	0.01	0.01	0.00	0.63	0.00	0.01	0.01
		Native Perennial forbs cover	0.02	0.00	0.13	0.00	0.00	0.00	0.00	0.13	0.00	0.00	0.00
		Native Annual forbs cover	0.02	0.00	0.41	0.00	0.01	0.01	0.00	1.00	0.00	0.02	0.02
		Non-native (annual herbaceous) species cover	0.02	0.00	0.70	0.00	0.01	0.01	0.00	0.70	0.00	0.01	0.01
		Leaf litter cover	0.02	0.00	0.71	0.00	0.01	0.01	0.00	0.71	0.00	0.01	0.01
		Woody debris (< 10 cm diameter) cover	0.02	0.00	0.36	0.00	0.01	0.01	0.00	0.36	0.00	0.01	0.01
		Logs (> 10 cm diameter) cover	0.02	0.00	0.03	0.00	0.00	0.00	0.00	0.03	0.00	0.00	0.00
		Logs (> 10 cm diameter) volume	0.02	0.00	0.02	0.00	0.00	0.00	0.00	0.02	0.00	0.00	0.00
	Functional state	*Soil*											
		Soil phosphorus availability	0.01	0.00	0.00	0.00	0.00	0.00	0.00	0.00	0.00	0.00	0.00
		Soil ammonium availability (NH4)	0.01	0.00	0.75	0.00	0.01	0.01	0.00	0.75	0.00	0.01	0.01
		Soil nitrate availability (NO3)	0.01	0.00	0.53	0.00	0.01	0.01	0.00	1.00	0.00	0.01	0.01
		Soil potassium availability	0.01	0.00	0.45	0.00	0.01	0.01	0.00	0.45	0.00	0.01	0.01
		Decomposition (green tea)	0.01	0.00	0.87	0.00	0.01	0.01	0.00	0.87	0.00	0.01	0.01
		Decomposition (roiboos tea)	0.01	0.00	0.32	0.00	0.00	0.00	0.00	0.32	0.00	0.00	0.00
		Decomposition (wood)	0.01	0.00	0.21	0.00	0.00	0.00	0.00	0.21	0.00	0.00	0.00
		Invertebrates (ants)											
		Dominant Dolichoderinae - abundance	0.01	0.00	0.00	0.00	0.00	0.00	0.00	1.00	0.00	0.01	0.01
		Dominant Dolichoderinae - richness	0.01	0.00	0.83	0.00	0.01	0.01	0.00	0.83	0.00	0.01	0.01
		Generalized Myrmicinae - abundance	0.01	0.00	0.54	0.00	0.01	0.01	0.00	0.00	0.00	0.00	0.00
		Generalized Myrmicinae - richness	0.01	0.00	0.88	0.00	0.01	0.01	0.00	1.00	0.00	0.01	0.01
		Opportunists - abundance	0.01	0.00	0.00	0.00	0.00	0.00	0.00	0.00	0.00	0.00	0.00
		Opportunists - richness	0.01	0.00	0.00	0.00	0.00	0.00	0.00	0.00	0.00	0.00	0.00
		Subordinate Camponotini - abundance	0.01	0.00	0.30	0.00	0.00	0.00	0.00	0.30	0.00	0.00	0.00
		Subordinate Camponotini - richness	0.01	0.00	0.56	0.00	0.01	0.01	0.00	0.56	0.00	0.01	0.01
		Hot-Climate Specialists - abundance	0.01	0.00	0.40	0.00	0.00	0.00	0.00	0.40	0.00	0.00	0.00
	Total biotic					0.00	0.24	0.24			0.00	0.26	0.26
Total			1.00			0.00	0.46	0.46			0.00	0.50	0.50

Table S4 Ecosystem condition index account continued (*Part 2 of 2*)

ADJUSTED INDICATOR WEIGHT										
	NOT TRUNCATED					TRUNCATED TO REFERENCE SYSTEM				
Indicator weight - adj	Indicator values (0 - 1)		Index values			Indicator values (0 - 1)		Index values		
	Opening	Closing	Opening	Closing	Change*	Opening	Closing	Opening	Closing	Change*
(4.2)	(5.3)	(6.3)	(7.3)	(8.3)	(9.3)	(5.4)	(6.4)	(7.4)	(8.4)	(9.4)
0.04	0.00	0.57	0.00	0.02	0.02	0.00	0.57	0.00	0.02	0.02
0.04	0.00	0.87	0.00	0.03	0.03	0.00	1.00	0.00	0.04	0.04
0.04	0.00	0.91	0.00	0.04	0.04	0.00	0.91	0.00	0.04	0.04
0.04	0.00	0.33	0.00	0.01	0.01	0.00	0.33	0.00	0.01	0.01
0.04	0.00	0.00	0.00	0.00	0.00	0.00	1.00	0.00	0.04	0.04
0.03	0.00	0.78	0.00	0.02	0.02	0.00	0.00	0.00	0.00	0.00
0.07	0.00	0.42	0.00	0.03	0.03	0.00	0.42	0.00	0.03	0.03
0.02	0.00	0.90	0.00	0.02	0.02	0.00	0.90	0.00	0.02	0.02
0.02	0.00	0.52	0.00	0.01	0.01	0.00	1.00	0.00	0.02	0.02
0.02	0.00	0.78	0.00	0.02	0.02	0.00	0.78	0.00	0.02	0.02
0.02	0.00	0.00	0.00	0.00	0.00	0.00	0.00	0.00	0.00	0.00
0.02	0.00	0.68	0.00	0.01	0.01	0.00	0.00	0.00	0.00	0.00
			**0.00**	**0.22**	**0.22**			**0.00**	**0.24**	**0.24**
0.05	0.00	0.00	0.00	0.00	0.00	0.00	0.00	0.00	0.00	0.00
0.02	0.00	1.00	0.00	0.02	0.02	0.00	1.00	0.00	0.02	0.02
0.02	0.00	0.59	0.00	0.01	0.01	0.00	0.59	0.00	0.01	0.01
0.02	0.00	0.00	0.00	0.00	0.00	0.00	0.00	0.00	0.00	0.00
0.02	0.00	0.23	0.00	0.00	0.00	0.00	0.23	0.00	0.00	0.00
0.02	0.00	0.39	0.00	0.01	0.01	0.00	0.00	0.00	0.00	0.00
0.01	0.00	0.34	0.00	0.00	0.00	0.00	0.34	0.00	0.00	0.00
0.02	0.00	0.00	0.00	0.00	0.00	0.00	0.00	0.00	0.00	0.00
0.02	0.00	0.78	0.00	0.02	0.02	0.00	0.78	0.00	0.02	0.02
0.02	0.00	0.63	0.00	0.01	0.01	0.00	0.63	0.00	0.01	0.01
0.02	0.00	0.54	0.00	0.01	0.01	0.00	0.54	0.00	0.01	0.01
0.02	0.00	0.63	0.00	0.01	0.01	0.00	0.63	0.00	0.01	0.01
0.02	0.00	0.13	0.00	0.00	0.00	0.00	0.13	0.00	0.00	0.00
0.02	0.00	0.41	0.00	0.01	0.01	0.00	1.00	0.00	0.02	0.02
0.05	0.00	0.70	0.00	0.03	0.03	0.00	0.70	0.00	0.03	0.03
0.01	0.00	0.71	0.00	0.01	0.01	0.00	0.71	0.00	0.01	0.01
0.01	0.00	0.36	0.00	0.00	0.00	0.00	0.36	0.00	0.00	0.00
0.01	0.00	0.03	0.00	0.00	0.00	0.00	0.03	0.00	0.00	0.00
0.01	0.00	0.02	0.00	0.00	0.00	0.00	0.02	0.00	0.00	0.00
0.01	0.00	0.00	0.00	0.00	0.00	0.00	0.00	0.00	0.00	0.00
0.01	0.00	0.75	0.00	0.01	0.01	0.00	0.75	0.00	0.01	0.01
0.01	0.00	0.53	0.00	0.01	0.01	0.00	1.00	0.00	0.01	0.01
0.01	0.00	0.45	0.00	0.01	0.01	0.00	0.45	0.00	0.01	0.01
0.01	0.00	0.87	0.00	0.01	0.01	0.00	0.87	0.00	0.01	0.01
0.01	0.00	0.32	0.00	0.00	0.00	0.00	0.32	0.00	0.00	0.00
0.01	0.00	0.21	0.00	0.00	0.00	0.00	0.21	0.00	0.00	0.00
0.01	0.00	0.00	0.00	0.00	0.00	0.00	1.00	0.00	0.01	0.01
0.01	0.00	0.83	0.00	0.01	0.01	0.00	0.83	0.00	0.01	0.01
0.01	0.00	0.54	0.00	0.01	0.01	0.00	0.00	0.00	0.00	0.00
0.01	0.00	0.88	0.00	0.01	0.01	0.00	1.00	0.00	0.01	0.01
0.01	0.00	0.00	0.00	0.00	0.00	0.00	0.00	0.00	0.00	0.00
0.01	0.00	0.00	0.00	0.00	0.00	0.00	0.00	0.00	0.00	0.00
0.01	0.00	0.30	0.00	0.00	0.00	0.00	0.30	0.00	0.00	0.00
0.01	0.00	0.56	0.00	0.01	0.01	0.00	0.56	0.00	0.01	0.01
0.01	0.00	0.40	0.00	0.00	0.00	0.00	0.40	0.00	0.00	0.00
			**0.00**	**0.24**	**0.24**			**0.00**	**0.26**	**0.26**
1.00			**0.00**	**0.46**	**0.46**			**0.00**	**0.49**	**0.49**

References

1. D. A. Keith *et al.*, A function-based typology for Earth's ecosystems. *Nature* **610**, 513-518 (2022).
2. Department of Climate Change, Energy, the Environment and Water, *Eucalypt Woodlands of the Western Australian Wheatbelt in Community and Species Profile and Threats Database,* https://www.dcceew.gov.au/environment/biodiversity/threatened/nominations/comment/wa-wheatbelt-woodlands, accessed 18 March 2024, (Canberra, 2020).
3. T. Parkhurst, R. Standish, S. Prober, P is for persistence - Soil phosphorus remains elevated for more than a decade after old field restoration. *Ecol. Appl.* **32**, (2021).
4. T. Parkhurst, R. J. Standish, S. M. Prober, Limited recovery of soil organic carbon and soil biophysical functions after old field restoration in an agricultural landscape. *Austral Ecology* **49**, e13519 (2024)
5. T. Parkhurst, S. M. Prober, R. J. Standish, Recovery of woody but not herbaceous native flora 10 years post old-field estoration. *Ecological Solutions and Evidence* **2**, e12097 (2021).
6. T. Parkhurst, R. J. Standish, A. N. Andersen, S. M. Prober, Old-field restoration improves habitat for ants in a semi-arid landscape. *Restoration Ecology*, e13605 (2021).
7. United Nations, European Commission, Food and Agriculture Organization, Organisation for Economic Co-operation and Development, World Bank, "System of Environmental-Economic Accounting—Ecosystem Accounting (SEEA EA). White cover publication.," https://seea.un.org/ecosystem-accounting, accessed 18 March 2024, (New York, 2021).
8. A. Andersen, A classification of Australian ant communities, based on functional groups which parallel plant life-forms in relation to stress and disturbance. *Journal of biogeography*, 15-29 (1995).
